# Complete Genome Sequence of the *Campylobacter ureolyticus* Clinical Isolate RIGS 9880

**DOI:** 10.1128/genomeA.01291-15

**Published:** 2015-11-05

**Authors:** William G. Miller, Emma Yee, Stephen L. W. On, Leif P. Andersen, James L. Bono

**Affiliations:** a Produce Safety and Microbiology Research Unit, Agricultural Research Service, U.S. Department of Agriculture, Albany, California, USA; b Institute of Environmental Science and Research Limited, Christchurch Science Centre, Christchurch, New Zealand; c Department of Clinical Microbiology, Copenhagen University Hospital, Rigshospitalet, Copenhagen, Denmark; d Meat Safety and Quality Research Unit, Agricultural Research Service, U.S. Department of Agriculture, Clay Center, Nebraska, USA

## Abstract

The emerging pathogen *Campylobacter ureolyticus* has been isolated from human and animal genital infections, human periodontal disease, domestic and food animals, and from cases of human gastroenteritis. We report the whole-genome sequence of the human clinical isolate RIGS 9880, which is the first closed genome for *C. ureolyticus*.

## GENOME ANNOUNCEMENT

The emerging organism *Campylobacter ureolyticus* (formerly *Bacteroides ureolyticus* [1]) was initially isolated from superficial soft tissue lesions, and genital and periodontal infections (2–4). However, *C. ureolyticus* has been increasingly isolated from cases of human gastroenteritis (5), including patients with ulcerative colitis (6) and Crohn’s disease (7). *C. ureolyticus* strain RIGS 9880 was isolated from a fecal sample from an immunocompromised patient with diarrhea at a tertiary hospital in Denmark. In this study, we report the whole-genome sequence of strain RIGS 9880, which represents the first closed *C. ureolyticus* genome.

Three next-generation sequencing platforms were used to complete the *C. ureolyticus* strain RIGS 9880 genome: Roche GS-FLX, Illumina HiSeq, and PacBio RS. A total of 533,763 shotgun and paired-end Roche 454 reads (101× coverage) were assembled, using the Roche Newbler assembler version 2.6, into a single scaffold of 12 contigs. Sanger DNA sequencing of contig-bridging amplicons was used to close the scaffold into a single contig. All 454 base calls were validated using 20,908,072 Illumina HiSeq reads (SeqWright, Houston, TX, USA), adding a coverage of 1,237×. An optical restriction map (OpGen, Gaithersburg, MD, USA) with restriction enzyme XbaI was used to validate the assembly. Finally, the domain structure of a large hypervariable adhesin-encoding gene was confirmed with PacBio Continuous Long Reads, adding a further 502× coverage (1,840× total coverage).

*C. ureolyticus* strain RIGS 9880 has a circular genome of 1,642 kb with a GC content of 29.23%. Protein-, rRNA- and tRNA-encoding genes were identified as described previously (8). The genome encodes 1,595 putative protein-coding genes, 24 pseudogenes, and 3 rRNA operons. No plasmids or hypervariable homopolymeric GC tracts (≥8 bp) were identified.

The RIGS 9880 genome contains several genes and features identified previously in *C. ureolyticus* (9), including a zonula occludens toxin genomic island (10, 11) and a large YadA-domain adhesin (9). In strain RIGS 9880, this adhesin gene comprises unique N- and C-terminal regions flanking a central region composed of tandemly repeated 203- and 460-bp domains. PCR amplification and PacBio sequencing indicates that this central region is hypervariable: within a population, genes are present containing a range of repeats, from a single 203-bp domain to 14½ 203-bp/460-bp domain pairs.

Consistent with the aflagellate nature of this species, strain RIGS 9880 does not contain genes encoding flagellar, flagellar modification, or chemotaxis proteins. Another noteworthy feature of strain RIGS 9880 is the absence of key glutamine amidotransferases (HisF, CarA, TrpG) related to amino acid (histidine, tryptophan, arginine) and pyrimidine biosynthesis. It is possible that *C. ureolyticus* uses ammonia derived from urease activity directly in these reactions. The pyrimidine biosynthetic pathway of *C. ureolyticus* strain RIGS 9880 is also unusual in that the PyrE and PyrF enzymes form a clade that is evolutionarily distinct from orthologs encoded by the remainder of *Campylobacter*. The most similar orthologs to *C. ureolyticus* PyrE and PyrF are those encoded by members of the *Veillonellaceae*. These features were identified also in the draft genome of the *C. ureolyticus* strain ACS-301-V-Sch3b (data not shown), suggesting that they are general characteristics of *C. ureolyticus*.

### Nucleotide sequence accession number.

The complete genome sequence of *C. ureolyticus* strain RIGS 9880 has been deposited in GenBank under the accession number CP012195.
